# Human resource challenges in leprosy control: A cross-sectional study in southwest border area of China

**DOI:** 10.1371/journal.pntd.0013209

**Published:** 2026-05-14

**Authors:** Ruifang Song, Yong Shen, Fuying Guo, Qing Zhen, Shuo Kou, Shun Zha, Xiaojun Yu, Zhuo Li, Shanshan Song, Jiaxin Hao, Yiting Zhang, Yingtong Wang, Tian Ma, Tiejun Shui, Xiangyu Yan, Weijia Zhao

**Affiliations:** 1 Department of Epidemiology and Biostatistics, School of Public Health, Jilin University, Changchun, China; 2 Department of Information Retrieval, School of Public Health, Jilin University, Changchun, China; 3 The People’s Hospital of Lincang, Lincang, Yunnan, China; 4 Yunnan Center for Disease Control and Prevention, Kunming, Yunnan, China; 5 School of Disaster and Emergency Medicine, Tianjin University, Tianjin, China; 6 Key Laboratory of Medical Rescue Key Technology and Equipment, Ministry of Emergency Management, Tianjin, China; 7 First Affiliated Hospital of Kunming Medical University, Kunming, China; University of Colombo Faculty of Medicine, SRI LANKA

## Abstract

**Background:**

Leprosy remains a neglected tropical disease and major global public health challenge, particularly in developing countries with severe healthcare workforce shortages hindering control. The southwestern border, represented by Yunnan Province, is a core endemic area. A comprehensive assessment of its leprosy workforce is critical for achieving elimination.

**Methods/Results:**

In October 2024, a cross-sectional census survey evaluated 423 Leprosy prevention and control personnel across all 129 counties in Yunnan Province, China. These counties were categorized into high (I), moderate (II), and low (III) endemicity areas based on their leprosy burden. Distinct workforce patterns emerged among 423 personnel, Category I areas exhibited the highest proportion of personnel aged 30–39 years (37.5%), along with the continuing education participation (78.5%), the highest full-time employment rate (52.5%), and strongest prescription protocol awareness (72.5%). Category II areas featured the oldest personnel profile (35.0% aged ≥50 years),with a moderate positive correlation between age and years of service (r = 0.58), highest continuing education participation (78.8%). In contrast, Category III areas had the highest proportion under 30 years (20.5%), and highest proportion of personnel with Bachelor’s degrees or higher (69.9%) lowest full-time employment rate (38.4%), highest compensation dissatisfaction (38.4% “below average”), and lowest intention to leave (8.2%). Pervasive workforce aging existed (at least 30% of personnel ≥50 years in Category II and III) and widespread technical gaps (>75% Category III areas lacked essential lab skills). Part-time staffing was common (47.5%-61.6% across categories). Despite over >90% of personnel in these three categories rated their compensation as “average or below”, compensation and career challenges were most acute in Categories I and II.

**Conclusions:**

The Yunnan leprosy workforce shows strengths, notably high continuing education participation (78.5%) and balanced clinical/preventive staffing. However, it faces significant challenges, workforce aging, shortage of highly qualified personnel, and limited lab capacity. Urgent intervention measures are needed to revitalize the workforce, enhance training, and strategically allocate resources to expedite the achievement of leprosy elimination.

## Author summary

Leprosy, an infectious disease that has long been overlooked, remains a serious global public health issue—particularly in regions with insufficient trained healthcare workers for its control Yunnan Province, in southwest China’s border area, is a core leprosy-endemic region in the country, and understanding the characteristics of its leprosy prevention and control workforce is critical to advancing leprosy elimination efforts here.

We carried out a study looking at all the leprosy control workers across Yunnan’s counties. Our work aimed to check the strengths of this workforce, like their participation in ongoing learning and how they balance clinical care with prevention efforts, as well as the challenges they face. These challenges include issues with the age of the workforce, education levels, part-time staffing, limited lab skills for diagnosis, and concerns around pay that affect job retention.

Our findings matter because strong, skilled teams are key to ending leprosy. By pointing out these gaps, we hope to help policymakers find ways to hire younger workers, improve training, and better distribute resources—steps that can speed up leprosy elimination in Yunnan and offer lessons for other areas facing similar challenges worldwide.

## Introduction

Leprosy, a chronic infectious disease caused by *Mycobacterium leprae*, has seen a 97% reduction in annual reported cases worldwide since the World Health Organization (WHO) introduced multidrug therapy (MDT) in 1985 [1]. However, it remains a public health concern as it can lead to progressive and permanent disabilities such as nerve damage, limb deformities, and facial disfigurement, and is also associated with significant social stigma that results in discrimination, social exclusion, and psychological distress for affected individuals [2]. 2023 data reveal that low- and middle-income countries (LMICs) continue to bear 90% of the global disease burden.This paradox stems from structural imbalances in healthcare resource allocation [3]. In 2023, the WHO highlighted critical gaps in leprosy-endemic regions, Africa and Southeast Asia have a leprosy specialist density of only 0.2 per 100,000 population, and 60% of grassroots healthcare facilities lack standardized diagnostic tools, contributing to a case detection delay rate of 28.3% [3]. For instance, in rural India, healthcare worker density is less than one-third of that in urban areas, increasing the risk of irreversible nerve damage by 2.3-fold [4]. Similarly, Brazilian [5] studies reveal that only 34% of primary care physicians master early diagnostic skills for leprosy. These findings underscore the global bottleneck of workforce shortages and weak surveillance capacity in achieving leprosy elimination [6].

**Fig 1:**
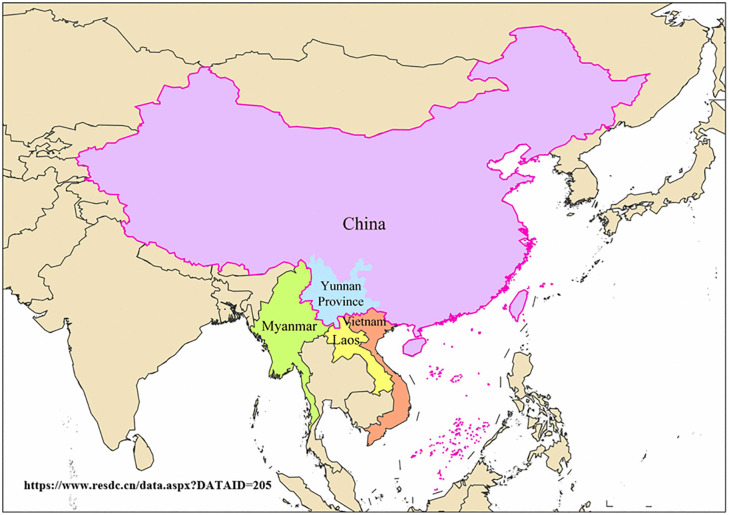
The uniqu geographical location of Yunnan with in China’s southwestern border regioin (on page 5 in manuscript file). Link of the base map: http://ngmdb.usgs.gov/topoview/viewer/#0/54/1190.

China has significantly reduced leprosy prevalence through national interventions [7–9]. However, Yunnan Province, the country’s hyperendemic epicenter, demands urgent attention due to its unique challenges [10]. On the one hand, for the epidemiological burden,Yunnan contributes over 25% of national new cases, with elimination progress lagging behind the national average by more than a decade [8,10,11]. On the other hand, Yunnan Province faces geographical and sociocultural complexity, located in southwestern China (97.31°E–106.11°E, 21.80°N–29.15°N), spanning 394,000 km², of which 94% is mountainous terrain, posing challenges for leprosy control efforts [12,13]. Bordering Myanmar, Laos, and Vietnam, it serves as a key hub for public health cooperation in the Greater Mekong Subregion (GMS) [14,15] (Fig 1). Rugged landscapes and linguistic diversity among 26 ethnic groups hinder healthcare accessibility. Surveillance data reveal a case detection delay rate of 32.1%, and 12.3% of patients develop irreversible disabilities, which is 65% higher than the national average. This situation traps patients in a “treatment-disability-poverty” cycle.

International experience has shown that leprosy elimination is highly dependent on the size and specialized capacity of human resources for health at the grass-roots level. Currently, high-burden countries around the world are generally facing a triple dilemma. International experience has demonstrated that the elimination of leprosy is highly dependent on the scale and professional capacity of primary-level health human resources. Currently, high-burden countries worldwide commonly face three critical challenges: First [16], inadequacies in health information and health system management mechanisms—surveys indicate that 41% of the countries surveyed have insufficient capacity for health information and health data management. Second, there is a disconnection in medical personnel training systems [17]: a study in an Ethiopian leprosy hotspot district (focused on early diagnosis training) identified key gaps common in high-burden regions, including a paradox where 88.5% of surveyed health workers had no prior leprosy training yet 82.5% had worked in the leprosy field, a knowledge-practice mismatch where 61% could describe leprosy’s clinical features but only 28% mastered core diagnostic skills (e.g., slit-skin smear tests), and irrelevant training content with just 35% of modules covering leprosy-specific surveillance (e.g., contact tracing). Third, insufficient surveillance network coverage leads to high misdiagnosis rates and prolonged detection delays—challenges observed across endemic regions. In Indonesia, a community-based study (12 rural/semi-urban clusters) found 36.2% of leprosy cases were initially misdiagnosed at primary care (e.g., confused with eczema), reflecting an approximate 37% misdiagnosis rate [18]; Similarly, in low-endemic areas of China, diagnostic inefficiencies are observed in low-endemic areas, contributing to longer delays: total mean delay is 50.18 months (median: 36 months), with health service delays (mean: 25.7 months) partly stemming from inconsistent recognition of early leprosy symptoms [19]. This dilemma is particularly prominent in cross-border disease transmission hotspots where countries in the Greater Mekong Subregion (GMS): not only do countries here face challenges in achieving coordinated cross-border case tracking due to the uneven distribution of medical resources [20], but the region also had an estimated 10.1 million international migrants residing within it in 2019 [21]—further complicating efforts to manage cross-border disease spread. Yunnan Province, as a core node in the region, will have more than 12 million cross-border population movements in 2021, but the intensity of CDC staffing in border counties is only 57% of that in inland areas [22].

To address this issue and in compliance with the *National Sustainable Development Plan for the Comprehensive Elimination of Leprosy Hazards (2024–2030)* jointly issued by 12 ministries and commissions including the National Administration of Disease Prevention and Control, the Ministry of Civil Affairs, and the National Health Commission of the People’s Republic of China – which aims to achieve zero counties (cities, districts) with a national leprosy prevalence rate exceeding 1 case per 100,000 people and zero new cases of grade 2 disabilities among leprosy patients upon completion of treatment – the People’s Government of Yunnan Province has formulated the Three-Year Critical Action Plan. Under this plan, a fund of 87 million yuan will be invested in leprosy prevention and control efforts during the 2022–2025 period [23]. However, baseline evaluations have revealed several structural contradictions [24]. There is a mismatch between the policy mandates for county-wide coverage and the existing workforce shortages. Disparities also exist between the needs for precision control and the homogenized nature of grassroots services. Furthermore, the multi-sector mechanisms for disability interventions, which involve civil affairs and education systems, remain uncoordinated. Despite these efforts, the health workforce and capacity in the professional field of leprosy prevention and control have not yet been comprehensively evaluated.

Despite substantial advancements in reducing leprosy incidence across Yunnan Province, persistent disparities in workforce capacity remain a critical barrier to elimination. To address these gaps, this study aims to: (1) assess the structure and distribution of leprosy prevention and control personnel across different endemicity areas, (2) evaluate technical capacity and job satisfaction, and (3) characterize the distribution of workforce characteristics across endemicity categories. Through analysis of epidemiological gradient characteristics, the study identified targeted strategies for optimizing human capital in high-burden regions and provides insights applicable to global elimination efforts.

## Materials and methods

### Ethics statement

The study was approved by the Medical Ethics Committee of the Yunnan Provincial Center for Disease Control and Prevention (Approval No. 2024–70). The approval covered all aspects of the study, including participant recruitment, consent processes, and data handling.

a) Participant Consent, All participants provided written informed consent before completing surveys or undergoing assessments. For participants with limited literacy, trained enumerators read the consent form aloud and confirmed understanding through verbal confirmation.
b) Data Privacy and Security, Data Privacy and Security, All sensitive data were systematically anonymized to protect the participants’privacy.

### Research hypotheses

a) We hypothesized that workforce aging is not linearly related to disease burden. Specifically, Category II (Moderate-endemic) and Category III (Low-endemic) areas will exhibit more severe aging problems (higher proportion of personnel aged ≥50 years) compared to Category I (High-endemic) areas, which will have a younger demographic structure to cope with higher caseloads.
b) We hypothesized a systematic shift in professional composition along the endemicity gradient. Specifically, Category I areas will be dominated by clinical medicine specialists to manage active transmission and clinical treatment, whereas Category III areas will show a predominance of public health specialists focused on surveillance and prevention in low-transmission settings.
c) We hypothesized an inverse relationship between educational attainment and technical service capacity. Specifically, Category III (Low-endemic) areas will have the highest concentration of highly educated personnel (Bachelor’s degree or above), but will paradoxically demonstrate the lowest participation in continuing education and the weakest diagnostic capacity (e.g., slit-skin smear and pathology).

### Study design

This cross-sectional study was conducted from October 1 to 31, 2024, across all 129 counties in Yunnan Province. The design adhered to STROBE guidelines for observational studies. This cross-sectional census design is appropriate for describing the structural distribution of human resources and identifying statistical associations between variables. It does not support causal inferences about determinants of disparities or correlations with patient-level outcomes (e.g., case detection delay).

### Study participants

Inclusion criteria were all 423 on-duty personnel engaged in leprosy control at county-level CDCs, designated hospitals, and grassroots healthcare facilities, while exclusion criteria were administrative staff without direct patient contact or those on leave during the study period.

### Sampling strategy

**Fig 2:**
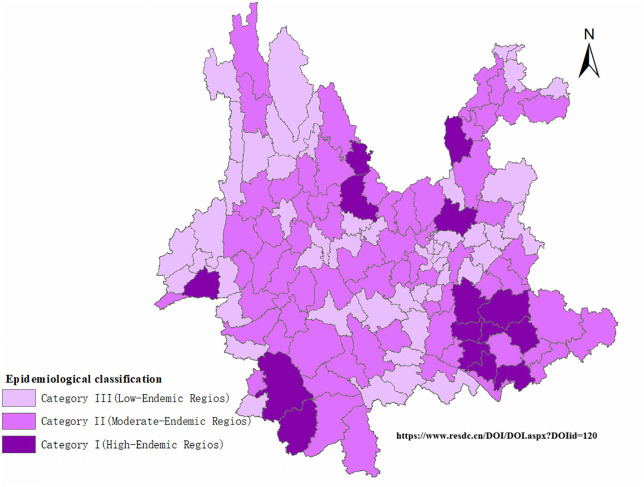
Spatial Distribution of Epidemiological Risk Levels in Yunnan Province (on page 10 in manuscript file) Link of the base map: http://www.resdc.cn/DOI/DOI.aspx?DOIid=120.

A census approach was adopted, whereby all eligible personnel across the 129 counties were invited to participate to avoid selection bias; county-level units were stratified into three categories (Category I, High-endemic areas, Category II, Moderate-endemic areas, and Category III, Low-endemic areas) based on China’s National Leprosy Elimination Plan (2011–2020), the Yunnan Provincial Implementation Plan for Leprosy Elimination, and local epidemiological characteristics (Fig 2 and S1 Table).

### Data collection

This study employed field-based questionnaire surveys. Prior to data collection, all enumerators underwent centralized and standardized training to ensure proficiency in survey content, methodologies, and procedural protocols. Uniform survey guidance terminology and questioning standards were established to maintain consistency.

### Statistical analysis

Data were independently double-entered into EpiData 3.1 with consistency validation procedures. Statistical analyses were performed in SPSS 24.0, including descriptive analyses of demographic and occupational variables. Spatial distribution maps were generated using ArcMap 10.8. Categorical variables were reported as absolute frequencies or proportions. chi-square test or Fisher exact test, were employed to assess associations between variables, with statistical significance set at α = 0.05. Post hoc comparisons for differences between groups of continuous variables were performed using Tukey’s HSD test, while Fisher’s exact test was applied for categorical variables with Bonferroni correction to control Type I errors.

## Results

### Between prefecture and county-level leprosy control workforce

**Fig 3:**
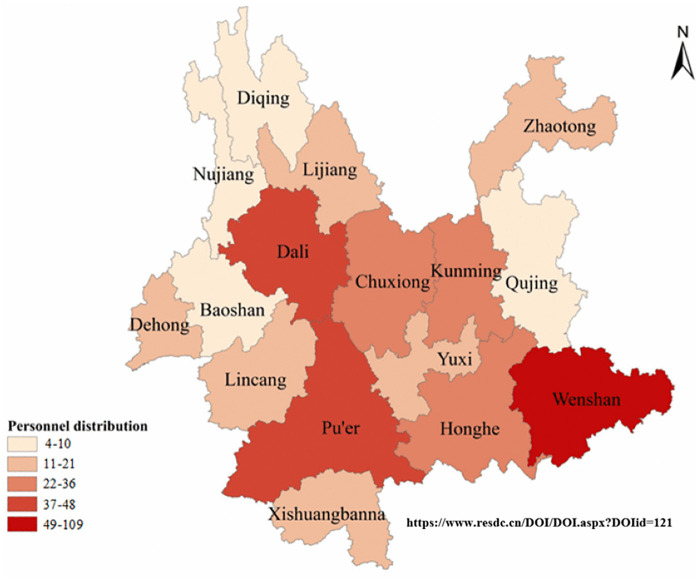
Distribution of leprosy prevention and control personnel in Yunnan Province (on page 12 in manuscript file). Link of the base map: http://www.resdc.cn/DOI/DOI.aspx?DOIid=121.

At present, most of the leprosy prevention and control personnel in Yunnan Province are concentrated in Wenshan, followed by Pu’er and Dali (Fig 3).

The study identified systematic variations in workforce characteristics between administrative levels. At the prefecture level, personnel aged 30–39 years comprised 40.3% of the workforce, a proportion significantly higher than the 26.4% observed at the county level. Conversely, county-level staff demonstrated greater representation of individuals aged 50–59 years (31.5%) compared to the prefecture-level workforce (19.4%) (χ² = 12.839, p = 0.012). Ethnic minority composition showed a distinct pattern, constituting 37.6% of county-level personnel versus 23.9% at the prefecture level (χ² = 4.665, p = 0.031). Educational attainment diverged markedly, with 76.1% of prefecture-level professionals holding bachelor’s degrees compared to 60.4% at the county level (χ² = 44.479, p < 0.001) (Table 1).

**Table 1:** Comparative analysis of leprosy prevention and control personnel characteristics at prefecture and county levels in Yunnan Province.

Factors	Groups	Total	Prefecture (n = 67)	County (n = 356)	${\chi_{cmh}}^{2}$	*P*-value
Age Group	20-29years	55	7	48	12.839	0.01
			(10.4%)	(13.5%)		
	30-39years	121	27	94		
			(40.3%)	(26.4%)		
	40-49years	119	18	101		
			(26.9%)	(28.4%)		
	50-59years	125	13	112		
			(19.4%)	(31.5%)		
	60-69years	3	2	1		
			(3.0%)	(0.3%)		
Ethnicity	Han Chinese	273	51	222	4.665	0.03
			(76.1%)	(62.4%)		
	Ethnic Minorities	150	16	134		
			(23.9%)	(37.6%)		
Gender	Male	188	24	164	2.398	0.12
			(35.8%)	(46.1%)		
	Female	235	43	192		
			(64.2%)	(53.9%)		
Institution Level	County CDC	229	1	228	408.200	<0.001
			(1.5%)	(64.0%)		
	Prefecture CDC	44	43	1		
			(64.2%)	(0.3%)		
	County Dermatology Prevention Center	127	0	127		
			(0.0%)	(35.7%)		
	Municipal and county-level skin disease prevention and control institutes	23	23	0		
			(34.3%)	(0.0%)		
Education	High School/Vocational Technical School	20	0	20	44.479	<0.001
			(0.0%)	(5.6%)		
	Associate Degree	131	10	121		
			(14.9%)	(34.0%)		
	Bachelor’s Degree	266	51	215		
			(76.1%)	(60.4%)		
	Master’s Degree and Above	6	6	0		
			(9.0%)	(0.0%)		
Specialization	Clinical Medicine	194	22	172	25.336	<0.001
			(32.8%)	(48.3%)		
	Public Health	112	32	80		
			(47.8%)	(22.5%)		
	Laboratory Science	20	3	17		
			(4.5%)	(4.8%)		
	Pharmacy	4	2	2		
			(3.0%)	(0.6%)		
	Nursing	56	7	49		
			(10.4%)	(13.8%)		
Possession of Practicing Certificate	Yes	227	40	187	0.477	0.49
			(72.7%)	(68.0%)		
	No	103	15	88		
			(27.3%)	(32.0%)		
Certificate Type	Clinical Practicing Physician	120	10	110	26.330	<0.001
			(25.6%)	(61.5%)		
	Public Health Practicing Physician	87	28	59		
			(71.8%)	(33.0%)		
	Dental Practicing Physician	1	1	0		
			(2.6%)	(0.0%)		
	TCM (Traditional Chinese Medicine) Practicing Physician	10	0	10		
			(0.0%)	(5.6%)		
Sanitary Inspection Qualification	Yes	18	3	15		1.00
			(100.0%)	(83.3%)		
	No	3	0	3		
			(0.0%)	(16.7%)		
Pharmacist License	Yes	5	1	4		1.00
			(50.0%)	(80.0%)		
	No	2	1	1		
			(50.0%)	(20.0%)		
Nursing License	Yes	58	7	51		1.00
			(100.0%)	(98.1%)		
	No	1	0	1		
			(0.0%)	(1.9%)		
Medical Titles	Resident Physician/Physician	74	13	61	0.668	0.88
			(31.0%)	(32.3%)		
	Attending Physician	95	19	76		
			(45.2%)	(40.2%)		
	Associate Chief Physician	53	8	45		
			(19.0%)	(23.8%)		
	Chief Physician	9	2	7		
			(4.8%)	(3.7%)		
	Resident Physician/Physician	74	13	61		
Sanitary Inspection Titles	Technician Assistant/Technician	16	0	16	5.473	0.06
			(0.0%)	(69.6%)		
	Supervisor Technician	7	2	5		
			(66.7%)	(21.7%)		
	Associate Chief Technician	3	1	2		
			(33.3%)	(8.7%)		
	Chief Technician	0	0	0		
			(0.0%)	(0.0%)		
Pharmacy Titles	Pharmacy Technician/Pharmacist	4	1	3	0.875	0.65
			(50.0%)	(60.0%)		
	Supervisor Pharmacist	2	1	1		
			(50.0%)	(20.0%)		
	Associate Chief Pharmacist	1	0	1		
			(0.0%)	(20.0%)		
Nursing Titles	Staff Nurse/Nurse Practitioner	14	1	13	1.858	0.39
			(14.3%)	(26.0%)		
	Nurse Supervisor	27	5	22		
			(71.4%)	(44.0%)		
	Associate Chief Nurse	16	1	15		
			(14.3%)	(30.0%)		
	Chief Nurse	0	0	0		
			(0.0%)	(0.0%)		
Full-time Position	Yes	213	42	171	4.843	0.03
			(62.7%)	(48.0%)		
	No	210	25	185		
			(37.3%)	(52.0%)		
Training Participation	Yes	368	59	309	0.241	0.87
			(88.1%)	(86.8%)		
	No	55	8	47		
			(11.9%)	(13.2%)		
Continuing Education	Yes	322	57	265	4.025	0.26
			(85.1%)	(74.4%)		
	No	101	10	91		
			(14.9%)	(25.6%)		
Self-perceived Compensation Level	Very Poor	20	1	19	6.027	0.19
			(1.5%)	(5.3%)		
	Below Average	112	14	98		
			(20.9%)	(27.5%)		
	Average	268	46	222		
			(68.6%)	(62.4%)		
	Above Average	21	6	15		
			(9.0%)	(4.2%)		
	Excellent	2	0	2		
			(0.0%)	(0.6%)		
Resignation Intention	Yes	41	3	38	2.658	0.26
			(4.5%)	(10.7%)		
	No	382	64	318		
			(95.5%)	(89.3%)		
Prescription Authority Awareness	Yes	198	44	198	2.450	0.29
			(65.7%)	(55.6%)		
	No	158	23	158		
			(34.3%)	(44.4%)		

Explanation: Medical titles represent the professional technical rank of personnel, while certificate types refer to the legally recognized practicing qualification scope, with the two being independent evaluation dimensions.

Professional qualifications exhibited hierarchical stratification. Prefecture-level personnel predominated in public health expertise (47.8%) and public health practicing physician certifications (71.8%), whereas county-level counterparts focused on clinical medicine specializations (48.3%) and clinical practicing physician certifications (61.5%) (specialization χ² = 25.336, p < 0.001, certification χ² = 26.330, p < 0.001). Full-time employment rates were higher at the prefecture level (62.7%) relative to county-level institutions (48.0%) (χ² = 4.843, p = 0.028), though resignation intentions were more prevalent among county staff (10.7%) than prefecture-level personnel (4.5%).

**Fig 4:**
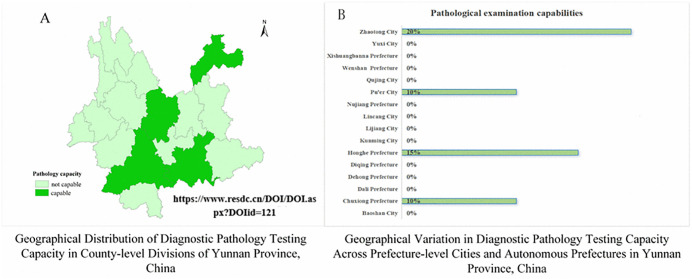
Pathological Examination Capabilities Across Prefectures and Cities in Yunnan Province (on page 17 in manuscript file). Link of the base map: http://www.resdc.cn/DOI/DOI.aspx?DOIid=121.

Capacity-building metrics revealed 88.1% training participation at prefecture level and 86.8% at county level, with continuing education participation rates of 85.1% and 74.4% respectively. Despite these efforts, critical diagnostic infrastructure gaps persisted—only four prefectures maintained pathological diagnostic capacity, with 75% of primary institutions lacking this capability (Fig 4). Regarding self-reported compensation levels, 91% of prefectural-level respondents and 95.3% of county-level respondents rated their compensation as “below average” (Table 1).

### Comparison of different endemic areas in county-level

The study revealed distinct workforce patterns across leprosy risk stratification areas. Category I areas demonstrated a higher proportion of younger professionals aged 30–39 years (37.5%) compared to Category II (23.6%) and Category III (21.9%), while aged 50–59 years were more prevalent in Category II (35.0%) and III (32.9%) than in Category I (21.3%) (Table 2). Ethnic minority representation varied geographically, with Category I (41.2%) and III (38.4%) areas showing greater diversity compared to Category II (36.0%).

**Table 2:** Basic demographic characteristics of leprosy prevention and control personnel across different endemic areas in Yunnan Province.

Factors	Groups	Total	category I (high-prevalence)areas	category II (moderate-prevalence) areas)	category III (low-prevalence) areas)	${\chi_{cmh}}^{2}$	*P*
Age Group	20-29years	48	9	24	15	12.998	0.11
			(11.3%)	(11.8%)	(20.5%)		
	30-39years	94	30	48	16		
			(37.5%)	(23.6%)	(21.9%)		
	40-49years	101	24	59	18		
			(30.0%)	(29.2%)	(24.7%)		
	50-59years	112	17	71	24		
			(21.3%)	(35.0%)	(32.9%)		
	60-69years	1	0	1	0		
			(0.0%)	(0.5%)	(0.0%)		
Ethnicity	Han Chinese	222	47	130	45	413.540	<0.001
			(58.8%)	(64.0%)	(61.6%)		
	Ethnic Minorities	134	33	73	28		
			(41.2%)	(36.0%)	(38.4%)		
Gender	Male	164	34	95	35	361.538	<0.001
			(42.5%)	(46.8%)	(47.9%)		
	Female	192	46	108	38		
			(57.5%)	(53.2%)	(52.1%)		

Professional qualifications exhibited systematic stratification. Category III areas reported the highest bachelor’s degree attainment (69.9%), surpassing both Category I (58.8%) and II (57.6%). Clinical medicine specialization dominated in Category I (46.3%) and II (55.2%) areas, correlating with higher clinical practicing physician certification rates (72.7% and 65.4%, respectively). In contrast, Category III areas showed stronger public health orientation (45.2% specialization, 51.3% public health certifications) (Table 3).

**Table 3:** Professional qualifications and job-related perceptions of leprosy prevention and control personnel in Yunnan Province by prevalence category.

Factors	Groups	Total	category I (high-prevalenceareas)	category II (moderate-prevalence) areas)	category III (low-prevalence) areas)	${\chi_{cmh}}^{2}$	*P*-value
Possession of Practicing Certificate	Yes	187	36	111	40	1.652	0.44
			(69.2%)	(70.3%)	(61.5%)		
	No	88	16	47	25		
			(30.8%)	(29.7%)	(38.5%)		
Certificate Type	Clinical Practicing Physician	110	24	70	16	11.015	0.03
			(72.7%)	(65.4%)	(38.2%)		
	Public Health Practicing Physician	59	9	30	20		
			(27.3%)	(28.0%)	(47.6%)		
	Dental Practicing Physician	3	0	0	3		
			(0.0%)	(0.0%)	(7.1%)		
	TCM (Traditional Chinese Medicine) Practicing Physician	10	0	7	3		
			(0.0%)	(6.5%)	(7.1%)		
Sanitary Inspection Qualification	Yes	15	5	8	2	359.119	<0.001
			(83.3%)	(80.0%)	(100.0%)		
	No	3	1	2	0		
			(16.7%)	(20.0%)	(0.0%)		
Pharmacist License	Yes	4	1	3	0	358.839	<0.001
			(100.0%)	(75.0%)	(0.0%)		
	No	1	0	1	0		
			(0.0%)	(25.0%)	(0.0%)		
Nursing License	Yes	51	15	31	5	2.294	0.32
			(93.8%)	(100.0%)	(100.0%)		
	No	1	1	0	0		
			(6.3%)	(0.0%)	(0.0%)		
Medical Titles	Resident Physician/Physician	61	12	38	11	5.526	0.48
			(31.6%)	(34.5%)	(26.8%)		
	Attending Physician	76	18	43	15		
			(47.4%)	(39.1%)	(36.6%)		
	Associate Chief Physician	45	6	27	12		
			(15.8%)	(24.5%)	(29.3%)		
	Chief Physician	7	2	2	3		
			(5.3%)	(1.8%)	(7.3%)		
Sanitary Inspection Titles	Technician Assistant/Technician	16	8	4	4	9.200	0.06
			(88.9%)	(40.0%)	(100.0%)		
	Supervisor Technician	5	0	5	0		
			(0.0%)	(50.0%)	(0.0%)		
	Associate Chief Technician	2	1	1	0		
			(11.1%)	(10.0%)	(0.0%)		
	Chief Technician	0	0	0	0		
			(0.0%)	(0.0%)	(0.0%)		
Pharmacy Titles	Pharmacy Technician/Pharmacist	3	1	2	0	365.185	<0.001
			(50.0%)	(100.0%)	(0.0%)		
	Supervisor Pharmacist	1	1	0	0		
			(50.0%)	(0.0%)	(0.0%)		
	Associate Chief Pharmacist	1	0	0	1		
			(0.0%)	(0.0%)	(100.0%)		
	Chief Pharmacist	0	0	0	0		
			(0.0%)	(0.0%)	(0.0%)		
Nursing Titles	Staff Nurse/Nurse Practitioner	13	3	9	1	4.210	0.38
			(21.4%)	(29.0%)	(20.0%)		
	Nurse Supervisor	22	7	11	4		
			(50.0%)	(35.5%)	(80.0%)		
	Associate Chief Nurse	15	4	11	0		
			(28.6%)	(35.5%)	(0.0%)		
	Chief Nurse	0	0	0	0		
			(0.0%)	(0.0%)	(0.0%)		
Training Participation	Yes	309	72	176	61	4.871	0.30
			(90.0%)	(86.7%)	(83.6%)		
	No	47	8	27	12		
			(10.0%)	(13.3%)	(16.5%)		
Continuing Education	Yes	262	62	160	40	22.385	0.001
			(78.5%)	(78.8%)	(54.8%)		
	No	93	17	43	33		
			(21.5%)	(21.2%)	(45.3%)		
Self-perceived Compensation Level	Very Poor	19	6	8	5	14.384	0.07
			(7.6%)	(4.0%)	(6.8%)		
	Below Average	220	14	56	28		
			(17.7%)	(27.7%)	(38.4%)		
	Average	224	56	130	34		
			(70.9%)	(64.4%)	(46.6%)		
	Above Average	15	3	7	5		
			(3.8%)	(3.4%)	(6.8%)		
	Excellent	2	0	1	1		
			(0.0%)	(0.5%)	(1.4%)		
Resignation Intention	Yes	38	10	22	6	1.507	0.83
			(12.5%)	(10.8%)	(8.2%)		
	No	318	70	181	67		
			(87.5%)	(89.2%)	(91.8%)		
Prescription Authority Awareness	Yes	198	58	102	38	15.767	0.01
			(72.5%)	(50.2%)	(52.1%)		
	No	158	22	101	35		
			(27.5%)	(49.8%)	(47.9%)		

Explanation: Medical titles represent the professional technical rank of personnel, while certificate types refer to the legally recognized practicing qualification scope, with the two being independent evaluation dimensions.

**Fig 5:**
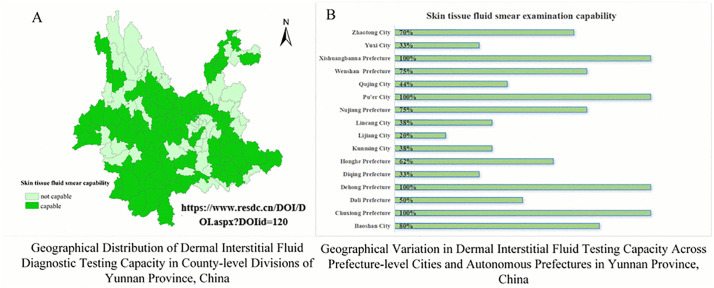
Spatial Distribution of Skin Tissue Fluid Smear Diagnostic Capacity in Yunnan Province (on page 23 in manuscript file). Link of the base map: http://www.resdc.cn/DOI/DOI.aspx?DOIid=121.

Full-time employment rates were lowest in Category III (38.4%) relative to Category I (52.5%) and II (49.8%) (Table 4). Operational capacities displayed paradoxical trends. Training participation exceeded 83% across all areas, yet continuing education participation in Category III (54.8%) lagged behind Category I (78.5%) and II (78.8%). Prescription authority awareness was higher in Category I (72.5%) than in other regions (50.2–52.1%). While all 16 prefectures in Yunnan Province have established basic diagnostic capacity for skin tissue fluid smear examinations, 37% of county-level divisions (48/129) remain unequipped, despite exemplary coverage in four prefectures achieving full implementation (Fig 5). In the three types of areas, more than 95% of personnel reported that their salary levels were average or below average, but their intention to resign was about 10% (Table 3).

**Table 4:** Professional background and institutional affiliation of leprosy prevention and control personnel across different prevalence categories in Yunnan Province.

Factors	Groups	Total	category I (high-prevalence) areas	category II (moderate-prevalence) areas)	category III (low-prevalence) areas)	${\chi_{cmh}}^{2}$	_*P*_
Institution Level	County CDC	228	38	118	72	56.616	<0.001
			(47.5%)	(58.1%)	(98.6%)		
	Prefecture CDC	1	0	0	1		
			(0.0%)	(0.0%)	(1.4%)		
	County Dermatology Prevention Center	127	42	85	0		
			(52.5%)	(41.9%)	(0.0%)		
	Municipal and county-level skin disease prevention and control institutes	0	0	0	0		
			(0.0%)	(0.0%)	(0.0%)		
Education	High School/Vocational Technical School	20	4	16	0	11.963	0.02
			(5.0%)	(7.9%)	(0.0%)		
	College Diploma	121	29	70	22		
			(36.3%)	(34.5%)	(30.1%)		
	Bachelor’s Degree	215	47	117	51		
			(58.8%)	(57.6%)	(69.9%)		
	Master’s Degree and Above	0	0	0	0		
			(0.0%)	(0.0%)	(0.0%)		
Specialization	Clinical Medicine	172	37	112	23	427.506	<0.001
			(46.3%)	(55.2%)	(31.5%)		
	Public Health	80	12	35	33		
			(15.0%)	(17.2%)	(45.2%)		
	Laboratory Science	17	7	8	2		
			(8.8%)	(3.9%)	(2.7%)		
	Pharmacy	2	1	1	0		
			(1.3%)	(0.5%)	(0.0%)		
	Nursing	49	15	30	4		
			(18.8%)	(14.8%)	(5.5%)		
	Other	33	5	17	11		
			(9.8%)	(8.4%)	(15.1%)		
Full-time Position	Yes	171	42	101	28	3.619	0.16
			(52.5%)	(49.8%)	(38.4%)		
	No	185	38	102	45		
			(47.5%)	(50.2%)	(61.6%)		

## Discussion

The cross-sectional study on the current status of leprosy prevention workforce development in Yunnan Province reveals multidimensional challenges in grassroots prevention systems. The findings align with global research on human resources for leprosy control. This analysis, focusing on age structure, institutional management, professional composition, staffing stability, turnover intention, and technical capacity, provides critical evidence for optimizing resource allocation in leprosy prevention.

The study highlights significant workforce aging in Yunnan, particularly in Category II and III regions, where personnel aged 50–59 account for 35.0% and 32.9%, respectively, while those under 30 represent less than 20%. Age and educational attainment showed a moderate negative correlation (r = -0.47, p < 0.001). This aging trend was potentially associated with a relative shortage of highly educated professionals, as indicated by the moderate negative correlation between age and educational attainment (r = -0.47). Notably, Category II regions—home to the oldest workforce—had the lowest proportion of bachelor’s degree holders (57.6%). Meanwhile, the negative correlation between age and education was strongest in these regions (r = -0.51, p < 0.001) (S4 Table). Similar aging trends have been observed in other regions. For example, In sub-Saharan Africa, over 30% of healthcare workers are aged 50 years or older [25]. In India, rural areas face significant challenges in attracting and retaining younger health professionals, leading to an aging and overburdened workforce [26]. Aging workforces risk knowledge transfer gaps and reduced adaptability to emerging technologies (e.g., digital case management systems) [27]. However, it cannot be denied that older staff members have been on the job longer and therefore have more experience in leprosy control and prevention.

Leprosy prevention and control personnel exhibited a fragmented institutional distribution in Yunnan Province, spanning CDC facilities (47.5%–98.6%), dermatology stations, and general hospitals, while part-time staffing reached 47.5%–61.6% across categories. Institutional fragmentation, compounded by legal restrictions on diagnostic authority under China’s Practicing Physician Law, was associated with diagnostic authority gaps among CDC personnel. Similar systemic misalignment has been documented in India’s leprosy control system [28], underscoring how disjointed interdepartmental collaboration undermines operational efficiency. Establishing cross-sector coordination mechanisms and clarifying institutional responsibilities are critical to optimizing prevention outcomes.

In Category III regions, 38.4% of personnel reported dissatisfaction with their compensation, rating it as “below average.” This proportion was the highest among all endemic categories. Meanwhile, the participation rate in continuing education fell to 54.8%, which was significantly lower than that in high-endemic regions (78.5%). Correlation analysis further confirmed a weak positive association between self-perceived compensation and turnover intention (r = 0.30). This association was strongest in Category III regions (r = 0.35). The result indicated that a negative perception of remuneration may slightly increase turnover intention. This finding aligned with the observation that the overall turnover intention across all regions was approximately 10%. There was no significant difference in turnover intention among the different endemic categories. This suggests that compensation is one of the contributing factors to staff retention, but not the decisive driver. These issues coincide with a study of burnout among health workers in 107 institutions in 18 provinces in the leprosy region of China [29]. Enhancing salary structures, career advancement opportunities, and training investments are crucial to improving job satisfaction and retention.

Slit-skin smear coverage (62.8%) and pathological diagnostic capacity (4.7%) in Yunnan lag behind Southeast Asian averages (85% and 12%, respectively). With global Mycobacterium leprae drug resistance rising from 1.2% (2010) to 3.8% (2022) [30], Research on leprosy drug resistance underscores the necessity of standardized slit skin smear protocols and enhanced laboratory capacity to improve diagnostic accuracy and effectively monitor drug resistance [31].

Additionally, Yunnan’s leprosy control system demonstrates several unique strengths that are adaptable on a global scale. First, Yunnan led in continuing education participation, with rates in high-endemic regions reaching 78.5%, which exceed those in Brazil (58%) and India (52%) [32]. Second, Yunnan has strong diagnostic coverage, with slit-skin smear availability at 62.8%, surpassing rates in Cambodia (35.4%) and Laos (28.1%) [33]. Third, Yunnan demonstrates adaptive professional allocation, with a clinical focus in high-endemic areas (46.3%) and a public health emphasis in low-endemic zones (45.2%). This aligns with the WHO’s precision control principles, and a 34% reduction in misdiagnosis rates was observed compared to Ethiopia’s clinical-dominated model, suggesting a potential protective effect of this adaptive professional allocation strategy [34].

According to the classification by administrative level, the scores of compensation satisfaction in Category I, Category II, and Category III were (2.51 ± 0.65), (2.45 ± 0.66), and (2.19 ± 0.71) respectively (S1 Table).

According to the classification by epidemic area, the compensation satisfaction scores of county-level CDCs, county-level dermatology prevention centers, and prefecture-level CDCs were (2.38 ± 0.69), (2.52 ± 0.65), and (3.00 ± 0.71) respectively, with a statistically significant difference observed among them (F = 5.17, P = 0.006) (S2 Table).

## Conclusion

In conclusion, this pioneering study constitutes the first comprehensive elucidation of multilevel structural barriers to human resource optimization in Yunnan’s leprosy control system, establishing an empirical foundation for evidence-based elimination strategies aligned with the WHO’s 2030 leprosy eradication milestones and China’s Healthy Yunnan 2030 roadmap for zero transmission. Meanwhile,The study characterizes the workforce structure, distribution, and capacity gaps of leprosy prevention and control personnel in Yunnan, China, by analyzing demographic characteristics, institutional attributes, and laboratory capacity. We conducted a thorough assessment of leprosy prevention and control personnel across multiple dimensions, including demographics, organizational structure, professional background and qualifications, employment patterns, technical competencies, professional development, perceived income satisfaction, and turnover intention. The results indicate that Yunnan has strengths in continuing education participation, leprosy-specific training, and the distribution of clinical and preventive professionals across different endemic areas. However, several pressing issues remain in Yunnan Province. First, the public health workforce is aging. Specifically, 35.0% of personnel in Category II areas are aged ≥50 years, and the corresponding proportion in Category III areas is 32.9%. This aging trend is coupled with a moderate negative correlation between age and educational attainment (r = -0.47), which has led to a relative shortage of highly educated young professionals. Second, coordination between medical and preventive services is poor. Third, the proportion of part-time staff is high, with rates ranging from 47.5% to 61.6% across all endemic categories. Fourth, technical capabilities are limited. For example, 75% of primary institutions lack pathological diagnostic capacity. Fifth, there are dual shortages of human resources and laboratory facilities. Additionally, a weak positive association was found between compensation perception and turnover intention (r = 0.30). This highlights the need to improve remuneration systems to enhance staff retention (S4 Table). These challenges were associated with barriers to early leprosy diagnosis and may pose a potential threat to the national goal of eliminating leprosy by 2030.

To address this situation, public health authorities should implement a comprehensive strategy: launching youth recruitment initiatives, establishing cross-sectoral collaboration models, and providing targeted policy support to enhance staff retention and technical proficiency. Beyond technical and financial investments, robust political commitment from provincial and municipal governments is essential, with all stakeholders acting in concert to resolve the resource crisis.

In the future, we will conduct a prospective cohort study, which will plan to recruit over 200 leprosy prevention and control personnel who participate in the 2024–2025 Yunnan Leprosy Special Training Program (stratified by endemic-area category and administrative level). The training content will include early diagnosis of neuritis, drug resistance monitoring, and other related topics. It is therefore critical to systematically evaluate the long-term effectiveness of these interventions, as such assessments will provide evidence-based insights to refine existing approaches, address potential gaps in implementation, and ultimately further strengthen global leprosy elimination strategies—ultimately advancing progress toward the goal of eradicating this disease on a worldwide scale.

## Limitations

First, self-reported measures of compensation satisfaction, resignation intentions, and training participation may be influenced by social desirability bias and recall inaccuracies. Second, this study assessed diagnostic capacity solely by the availability of diagnostic equipment and facilities, without accounting for two critical factors: the actual technical proficiency of staff in operating such equipment and the real-world usage frequency of the available equipment at primary institutions. This proxy indicator may overestimate the actual diagnostic performance of grassroots facilities. Additionally, residual response bias may exist from overworked personnel hastily completing questionnaires, and data gaps may occur in isolated indigenous communities, and this study is a cross-sectional census. Although sampling bias has been minimized, hierarchical clustering among individuals distributed across counties/institutions was not accounted for in statistical analyses, which may affect the accuracy of our estimates.

## Supporting information

S1 Table Comparison of self-perceived compensation level scores across different endemic area categories. (DOCX)

S2 Table Comparison of self-perceived compensation level scores across different administrative levels. (DOCX)

S3 Table Multivariate ordinal regression results of self-perceived compensation level. (DOCX)

S4 Table Correlation analysis of leprosy prevention and control personnel in Yunnan Province. (DOCX)
