# Supplementary material for: Human resource challenges in leprosy control: A cross-sectional study in southwest border area of China
Source: PLoS Negl Trop Dis. 2026 May 14;20(5):e0013209. doi: 10.1371/journal.pntd.0013209 (PMC13175470; doi:10.1371/journal.pntd.0013209)
Supplement: S1 Table — (DOCX) [file pntd.0013209.s001.docx]

**S1 Table Comparison of Self-perceived Compensation Level Scores Across Different Endemic Area Categories**

| **Endemic Area Category** | **Sample Size (n)** | **Score (Mean ± SD)** | **Median** | **Composition Ratio of Different Compensation Levels** |  |  |  |  |
| --- | --- | --- | --- | --- | --- | --- | --- | --- |
|  |  |  |  | Very Poor (Score 1) | Below Average (Score 2) | Average (Score 3) | Above Average (Score 4) | Excellent (Score 5) |
| Category I (High-endemic Area) | 80 | 2.51±0.65 | 3 | 7.5 **%**(6/80) | 17.5 (14/80) | 70.0 (56/80) | 3.8 (3/80) | 1.3 (1/80) |
| Category II (Medium-endemic Area) | 203 | 2.45±0.66 | 2 | 4.0 **%**(8/203) | 27.6 (56/203) | 64.0 (130/203) | 3.4 (7/203) | 0.5 (1/203) |
| Category III (Low-endemic Area) | 75 | 2.19±0.71 | 2 | 6.7 **%**(5/75) | 37.3 (28/75) | 48.0 (36/75) | 6.7 (5/75) | 1.3 (1/75) |
| Total | 358 | 2.43±0.68 | 2 | 5.3**%** (19/358) | 33.2 (119/358) | 62.6 (224/358) | 4.2 (15/358) | 1.7 (6/358) |
| *F* Value | - | 4.82 | - | - | - | - | - | - |
| *P* Value | - | 0.009 | - | - | - | - | - | - |
