# Supplementary material for: Human resource challenges in leprosy control: A cross-sectional study in southwest border area of China
Source: PLoS Negl Trop Dis. 2026 May 14;20(5):e0013209. doi: 10.1371/journal.pntd.0013209 (PMC13175470; doi:10.1371/journal.pntd.0013209)
Supplement: S2 Table — (DOCX) [file pntd.0013209.s002.docx]

**S2 Table Comparison of Self-perceived Compensation Level Scores Across Different Administrative Levels**

| **Administrative Level** | **Sample Size (n)** | **Score (Mean ± SD)** | **Median** | ***F* Value** | ***P* Value** |
| --- | --- | --- | --- | --- | --- |
| County-level CDC | 228 | 2.38±0.69 | 2 | 5.17 | 0.006 |
| County-level Institute of Dermatology and Venereology | 127 | 2.52±0.65 | 3 | - | - |
| Prefecture-level CDC | 3 | 3.00±0.71 | 3 | - | - |
