# Supplementary material for: Human resource challenges in leprosy control: A cross-sectional study in southwest border area of China
Source: PLoS Negl Trop Dis. 2026 May 14;20(5):e0013209. doi: 10.1371/journal.pntd.0013209 (PMC13175470; doi:10.1371/journal.pntd.0013209)
Supplement: S3 Table — (DOCX) [file pntd.0013209.s003.docx]

**S3 Table Multivariate Ordinal Regression Results of Self-perceived Compensation Level**

| **Variables** | **Groups (Reference Group)** | **Regression Coefficient (*β*)** | **Standard Error (SE)** | **Wald *χ²*** | **OR Value (95%CI)** | ***P* Value** |
| --- | --- | --- | --- | --- | --- | --- |
| Educational | Senior High School/Technical School | - | - | - | 1.00 | - |
|  | College Diploma | 0.28 | 0.15 | 3.47 | 1.32 (0.98-1.78) | 0.068 |
|  | Bachelor's Degree | 0.48 | 0.16 | 8.86 | 1.62 (1.18-2.23) | 0.003 |
| Professional Title | Junior (Resident Physician/Physician) | - | - | - | 1.00 | - |
|  | Intermediate (Attending Physician) | 0.35 | 0.18 | 3.76 | 1.42 (0.99-2.03) | 0.085 |
|  | Senior (Associate Chief Physician/Chief Physician) | 0.68 | 0.21 | 10.49 | 1.98 (1.35-2.90) | <0.001 |
| Continuing Education Participation | No | - | - | - | 1.00 | - |
|  | Yes | 0.45 | 0.18 | 6.25 | 1.57 (1.12-2.20) | 0.009 |
| Administrative Level (Adjusted Variable) | County CDC | - | - | - | 1.00 | - |
|  | County Dermatology Prevention Center | 0.26 | 0.15 | 3.01 | 1.30 (0.96-1.75) | 0.091 |
|  | Prefecture CDC | 0.40 | 0.19 | 4.41 | 1.49 (1.03-2.16) | 0.032 |
| Endemic-area Category (Adjusted Variable) | Category III (Low-prevalence) | - | - | - | 1.00 | - |
|  | Category II (Medium-prevalence) | 0.29 | 0.17 | 2.89 | 1.34 (0.95-1.89) | 0.098 |
|  | Category I (High-prevalence) | 0.32 | 0.18 | 3.17 | 1.38 (0.95-2.01) | 0.092 |
