# Supplementary material for: Human resource challenges in leprosy control: A cross-sectional study in southwest border area of China
Source: PLoS Negl Trop Dis. 2026 May 14;20(5):e0013209. doi: 10.1371/journal.pntd.0013209 (PMC13175470; doi:10.1371/journal.pntd.0013209)
Supplement: S4 Table — (DOCX) [file pntd.0013209.s004.docx]

**S4 Table Correlation Analysis of Leprosy Prevention and Control Personnel in Yunnan Province**

|  |  | Age | Education | Years of Service | Self-perceived Compensation | Resignation Intention |
| --- | --- | --- | --- | --- | --- | --- |
| All Areas | Age | 1.00 |  |  |  |  |
|  | Education | -0.47** | 1.00 |  |  |  |
|  | Years of Seevice | 0.58** | -0.40* | 1.00 |  |  |
|  | Self-perceived Compensation | 0.05 | -0.06 | 0.09 | 1.00 |  |
|  | Resignation Intention | 0.05 | -0.02 | 0.00 | 0.30* | 1.00 |
| Category I | Age | 1.00 |  |  |  |  |
|  | Education | -0.42* | 1.00 |  |  |  |
|  | Years of Seevice | 0.51* | -0.38* | 1.00 |  |  |
|  | Self-perceived Compensation | 0.04 | -0.05 | 0.09 | 1.00 |  |
|  | Resignation Intention | 0.03 | -0.03 | 0.08 | 0.22* | 1.00 |
| Category II | Age | 1.00 |  |  |  |  |
|  | Education | -0.51** | 1.00 |  |  |  |
|  | Years of Seevice | 0.62** | -0.43** | 1.00 |  |  |
|  | Self-perceived Compensation | 0.06 | -0.07 | 0.10 | 1.00 |  |
|  | Resignation Intention | 0.04 | -0.01 | 0.02 | 0.28* | 1.00 |
| Category III | Age | 1.00 |  |  |  |  |
|  | Education | -0.45** | 1.00 |  |  |  |
|  | Years of Seevice | -0.55 | -0.41** | 1.00 |  |  |
|  | Self-perceived Compensation | 0.55 | -0.08 | 0.07 | 1.00 |  |
|  | Resignation Intention | 0.07 | -0.04 | -0.01 | 0.35* | 1.00 |
